# Preliminary results on novel adjuvant combinations suggest enhanced immunogenicity of whole inactivated pandemic influenza vaccines

**DOI:** 10.3389/fddev.2024.1382266

**Published:** 2024-07-16

**Authors:** Allegra Peletta, Aurélie Marmy, Samo Guzelj, Alcidia Ramos Barros, Žiga Jakopin, Gerrit Borchard

**Affiliations:** ^1^ Section of Pharmaceutical Sciences, Institute of Pharmaceutical Sciences of Western Switzerland (ISPSO), University of Geneva, Geneva, Switzerland; ^2^ Faculty of Pharmacy, University of Ljubljana, Ljubljana, Slovenia

**Keywords:** pandemic influenza vaccine, adjuvant, hybrid PLGA nanoparticle, tomatine saponin, NOD2 agonists, formulation, immunogenicity

## Abstract

Due to the inherent risk of a further pandemic influenza outbreak, there is a need and growing interest in investigating combinations of prophylactic vaccines and novel adjuvants, particularly to achieve antigen dose sparing and improved immunogenicity. Influenza is a highly variable virus, where the specific vaccine target is constantly changing, representing a major challenge to influenza vaccine development. Currently, commercial inactivated influenza vaccines have a poor CD8^+^ T response, which impacts cross-reactivity and the duration of response. Adjuvanted influenza vaccines can increase immune responses, thereby achieving better protection and cross-reactivity to help contain the spread of the disease. An early exploration of a hybrid cholesterol-PLGA nanoparticle delivery system containing the saponin tomatine and a NOD2 (nucleotide-binding oligomerization domain 2) agonist called SG101 was conducted. This combination was preliminarily evaluated for its ability to induce cellular immunity when combined with whole inactivated virus (WIV) influenza vaccine. After the adjuvants were manufactured using a single emulsion process, two formulations with different drug loadings were selected and physico-chemically characterized, showing sizes between 224 ± 32 and 309 ± 45 nm and different morphologies. After ensuring the lack of *in vitro* toxicity and hemolytic activity, a pilot *in vivo* assay evaluated the hybrid nanoparticle formulation for its ability to induce humoral and cellular immunity when combined with whole inactivated virus (WIV) H5N1 influenza vaccine by intramuscular administration in mice. Hemagglutinin inhibition (HAI) titers for adjuvanted groups showed no significant difference compared to the group vaccinated with the antigen alone. It was similar for CD4^+^ and CD8^+^ T cell responses, although the high drug loading formulation induced higher titers of IFNγ-positive CD8^+^ T cells. These proof-of-concept results encourage further investigations to develop the hybrid formulation with increased or different loading ratios, to investigate manufacturing optimization, and to evaluate the role of the individual immunostimulatory compounds in immune responses.

## Graphical Abstract

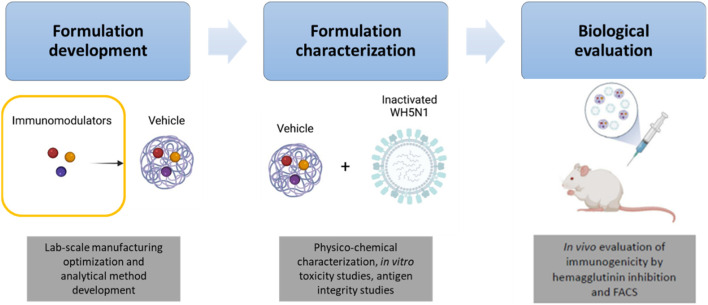

## 1 Introduction

Adjuvant research is of primary importance in the development of novel and safe vaccines for mutating infectious diseases. Pandemic influenza is a good example; in fact, researchers agree that the emergence of a new pandemic strain is not a question of *if* but a question of *where* and *when* (Harrington et al., 2021). The most efficient way of controlling the spread of a new pandemic virus is through the development of safe and effective vaccines in a short amount of time and with sufficient production capacity for the global population. In 2009, several vaccines against H1N1 pandemic influenza were developed to counter the effects of the pandemic. In one of those vaccines, the use of an adjuvant MF59^®^ allowed dose-sparing and increased humoral response. Unfortunately, the H1N1 vaccines never reached the majority of the world’s population in time, partly because of insufficient antigen production capacity, the necessity of multiple shots to achieve sufficient immunity, and long development times (Collin et al., 2009; Broadbent and Subbarao, 2011). Some of the reasons why unadjuvanted WIVs are considered for such vaccines are ease of manufacture, low reduced costs of production, and wide knowledge of the vaccine type (Wood and Robertson, 2004). Nevertheless, the use of appropriate adjuvants in combination with WIV vaccines can result in specific immune responses deemed important for cross-reactivity and durability. In particular, the role of CD8^+^ T cell-specific adjuvants has been highlighted in recent publications (Kumar et al., 2017; Clemens et al., 2018). There remains a need to identify new adjuvant technologies or combinations capable of inducing strong cellular response, which was shown to be a key requirement for immunity against influenza viruses (Clemens et al., 2018). Indeed, in previously approved pandemic vaccines adjuvanted with MF59^®^, AS03 (oil-in-water emulsions), or aluminum salts, CD8^+^ T-cell responses were not satisfactory (Atmar and Keitel, 2009; Leroux-Roels, 2009; Caillet et al., 2010; Cohet et al., 2019). Nanoparticles such as polymeric particles are employed to increase drug delivery. Adjuvant combinations ideally generate an additive or synergistic effect, resulting in adjuvant-sparing and reducing their risk of toxicity by reducing their therapeutic dose (Tom et al., 2019).

**FIGURE 1:**
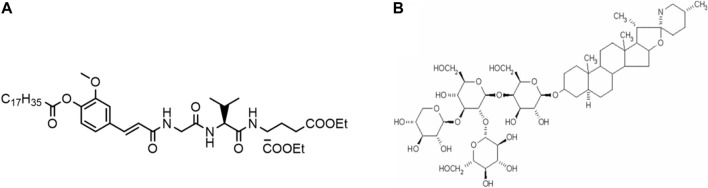
Chemical structures of **(A)** NOD2 receptor agonist SG101 and **(B)** tomatine.

A challenge for combinatory adjuvant formulations are their production and characterization. Both typically require empirical optimizations that combine important features of pharmaceutical drug delivery development and immunology to evaluate preclinical responses. We here report on the development and characterization of a combinatory adjuvant composed of two novel immunostimulatory compounds: the saponin tomatine and the small molecule SG101 (see structure in Figure 1A), a NOD2-like receptor (NLR) agonist encapsulated into PLGA-cholesterol (PLGA-Chol in the text) nanoparticles (NPs). The goal of this study is to investigate the potential *in vivo* activity of novel adjuvants not yet approved by regulatory agencies. These adjuvant formulations were then evaluated for immunological responses with a WIV H5N1 influenza vaccine in mice.

Tomatine (see structure in Figure 1B) is a saponin, more specifically a glycoalkaloid, that is found in tomatoes (Morrow et al., 2004). Like QS-21, a widely used Th 1-inducing vaccine adjuvant, tomatine has been shown to enhance immune response, particularly cytotoxic T cell activation (Heal and Taylor-Robinson, 2010). Tomatine induces permeabilization of the membrane of cells by interacting with cholesterol, resulting in a loss of cell content that leads to cell disruption (Yang et al., 2004). In order to overcome their toxicity, saponins are often formulated with cholesterol, which reduces their membrane permeabilization (Sun et al., 2009). Tomatine has been tested in a pre-clinical trial as an adjuvant in vaccine against pre-erythrocyte malaria (Heal and Taylor-Robinson, 2010).

NOD2 is an intracellular receptor, part of the NLRs family, which is expressed by various types of cells including macrophages and dendritic cells (DCs). This receptor recognizes peptidoglycan moieties such as muramyl dipeptide (MDP), a constituent of the bacterial cell wall, found in both gram-positive and -negative bacteria (Boyle et al., 2014). SG101 is a lipidated analog of MDP developed by the group of Professor Jakopin at the University of Ljubljana. Its high hydrophobicity increases its passage through the cell wall and facilitates its encapsulation into hydrophobic materials such as PLGA; it was shown to induce a balanced Th1/Th2 response *in vivo* (Guzelj et al., 2021).

The choice of combining saponins with NOD2 ligands arises from the desire to investigate the combinatory effect of an innate immune agonist, such as SG101, together with a saponin, inducing an adaptive immune response enhancement (Morrow et al., 2004; Marciani, 2018; Guzelj et al., 2021). Various such combinations of have already been investigated, such as saponins with TLR agonists (Ou et al., 2023). The investigation of novel combinatory adjuvants to elicit a broad and strong immune response with reduced doses is considered a pillar of modern vaccinology (Mount et al., 2013).

The choice of PLGA as a delivery system allows freeze-drying and the possibility of using a wide variety of manufacturing techniques as well as various solvents for formulation. Freeze-drying is of major importance for enhancing stability (Trenkenschuh and Friess, 2021). As the two adjuvants selected have a very different solubility profile, the testing of multiple solvents is a major advantage and permits identification of an ideal protocol for formulation. Last but not least, PLGA is an FDA-approved material, and its hydrophobicity as well as its molecular weight can be modulated to fit the encapsulated molecules (Operti et al., 2021). Cholesterol is necessary in this project as we need to quench the hemolytic activity of the saponin to avoid cytotoxicity (Wang et al., 2020).

## 2 Materials and methods

### 2.1 Materials

PLGA (Resomer^®^ RG 502H) with an average molecular weight of 7–17 kDa, cholesterol, α-tomatine, sucrose, and dichloromethane (DCM) were obtained from Sigma^®^ Aldrich (Buchs, Switzerland). SG101 was kindly provided by the University of Ljubljana, (Slovenia). Polyvinyl alcohol 8-88 (PVA) 67 kDa was supplied by Merck KGaA Emprove^®^ Essential (Darmstadt, Germany). Dulbecco’s Phosphate Buffered Saline (DPBS) was purchased from Gibco^®^ Thermo Fisher (Reinach, Switzerland). Monovalent bulk, egg-derived, β-propiolactone whole inactivated virus (WIV) A/turkey/Turkey/1/2005 (WH5N1) was kindly provided by the Vaccine Formulation Institute (Geneva, Switzerland). The defibrinated sheep blood for hemolysis assay was purchased from Oxoid Ltd. Thermo Fisher (Hampshire, UK).

### 2.2 Cell culture

Murine macrophage RAW 264.7 cell line was obtained from ATCC^®^ TIB-71 and cultured in Dulbecco’s modified Eagle’s medium (DMEM) completed with 10% fetal bovine serum (FBS), 0.1% penicillin/streptomycin, 1 mM sodium pyruvate, and 1 mM glutamine (Gibco, Thermo Fisher^®^) at 37 °C under a humidified atmosphere containing 5% CO_2_.

### 2.3 PLGA nanoparticle preparation

Two formulations of PLGA-Chol nanoparticles were manufactured using oil-in-water emulsion and solvent evaporation. In brief, 1 mL of DCM containing various amounts of PLGA, cholesterol, tomatine, and SG101 was added to 3 or 8 mL of 2% PVA solution and sonicated at an amplitude of 60% for 15 min using a probe sonicator (Branson Digital Sonifer^®^, USA). Two different samples were prepared: a low SG101 loading sample (FA: Formulation A) with 1.1 mg tomatine and 0.3 mg SG101 for 19.9 mg PLGA-Chol (at a 1:1 mass ratio of cholesterol:saponin) and a high SG101 loading sample (FB: Formulation B) with 3.5 mg tomatine and 0.5 mg SG101 for 12.9 mg PLGA-Chol. After sonication, DCM solvent was evaporated at room temperature (RT) under constant stirring for 2 h. Particles were centrifuged for 10 min at 4 °C and 22,000 rpm (Avanti™ 30 centrifuge, Beckman and Coulter, USA), and the supernatant was discarded. Samples were resuspended in a lyoprotectant solution composed of 2% w/v sucrose, then were frozen at −80°C for 2 h at atmosphere pressure. The other sample’s water was sublimated in a freeze-dryer at 0.1 mbar and shelf temperature of 4 °C, then dried for 3 days in the same conditions (Lyophilisator Alpha 1-2 LSC, Christ^®^, USA). NPs were characterized after resuspension in PBS or MilliQ water and vortexed 5 min at RT. Dynamic light scattering (DLS) (Zetasizer, Malvern, UK) was employed to assess the size and size distribution of the formulations. Nanoparticle morphology was evaluated by scanning electron microscopy (SEM) (JSM-700-1FA, JEOL, Japan). Freeze-dried nanoparticles were resuspended in 1 mL MilliQ water, then 10 μL of the particles’ suspension were placed on carbon tape and dried under vacuum at room temperature for 2–3 days. Analysis was performed at 5 kV voltage, WD = 10 mm. The paper reports the results of three distinct batches.

### 2.4 Determination of encapsulation efficacy by U-HPLC-MS

The tomatine and SG101 were quantified by U-HPLC-MS. Reported here are the results of three distinct batches. The instrument used was an ACQUITY Premier coupled to a SQD2 simple quadrupole mass spectrometer (Waters) using electrospray ionization (ESI). In brief, the compounds were extracted from the PLGA using a methanol–water 9:1 v/v mixture for 1 h under agitation. After centrifugation (10 min at 10′000 g), the supernatant was collected and diluted with a solution containing an internal standard (solasonine at 0.2 μg/mL in methanol/water 9:1 v/v). The reversed-phase LC separations were performed at 40 °C on a XSelect PREMIER Peptide HSS T3 2.5 µm (50 × 2.1 mm column) from Waters with an injection volume of 2 µL. The mobile phase was composed of 5 mM ammonium hydrogencarbonate buffer at pH 7 (phase A) and isopropanol (phase B) with a flow rate of 0.3 mL/min. The gradient’s parameters were 10%–80% phase B for 1 min, then 80% phase B for 3 min followed by column reconditioning at 10% phase B for 2 min. The temperature of the autosampler was 20 °C. Analyses were performed in positive ESI mode with selected ion monitoring (SIM) with a source temperature of 150 °C. The flow of desolvation gas (N_2_) was 1000 L/h at 450 °C and that of cone gas (N_2_) was 10 L/h. The capillary voltage was 3.0 kV. Adjuvant mass was extrapolated based on peak intensity. Drug loading and encapsulation efficacy were assessed with the following Eqs 1, 2

$$\%\;Drug\;Loading=\frac{\frac{Nanoparticles\;\left(mg\right)-sucrose\;\left(mg\right)}{1000}}{\left(Extrapolated\;adjuvant\;mass\;\left(µg\right)\right)},$$ (1)

$$\%\;Encapsulation\;Efficacy=\frac{\%\;Drug\;Loading\;X\;\left(Starting\;Nanoparticles\;mass\;\left(mg\right)-Sucrose\;\left(mg\right)\right)}{Starting\;adjuvant\;mass\;\left(mg\right)}.$$ (2)

### 2.5 WST-1 assay

WST-1 assay was performed to assess the biocompatibility of PLGA-Chol nanoparticles on RAW 264.7 cells. First, 1 × 10^5^ cells/mL were plated and cultured for 24 h in 100 μL supplemented DMEM (growth media) on a 96-well flat-bottom plate at 37 °C, 5% CO_2_. Next, the growth media was discarded and replaced with 100 μL nanoparticle suspension at different concentrations. Lyophilized PLGA-Chol nanoparticles were resuspended in Complete-DMEM (C-DMEM) at a stock solution concentration of 240 μg/mL of tomatine. Serial two-fold dilutions were carried out to obtain NP concentrations of 120, 60, and 30 μg/mL of tomatine. Samples with 103.4 μg/mL of tomatine, 32 μg/mL SG101 alone, or water for injection (WFI) were used as positive controls and untreated cells as a negative control. PLGA particles and controls were incubated on RAW 264.7 cells for 4 h at 37 °C, 5% CO_2_. After this step, 100 μL DMEM containing WST-1 reagent were added in each well and incubated for 1 h at 37 °C, 5% CO_2_. Absorbance was measured at 450 nm and 690 nm on a plate reader (SynergyMX, Agilent, USA). All data were normalized to the negative control for analysis. The experiment was repeated four times.

### 2.6 Hemolysis assay

Exactly 1 mL of DPBS 1x −/− was added to 200 µL of defibrinated sheep blood (Thermo Fisher, UK), then mixed and centrifuged at 1,530 rpm, 20 °C for 2 min. Supernatant was discarded, and the operation repeated until it was transparent. After the last wash, sheep blood was resuspended in 12 mL of DPBS. For the preparation of samples, tomatine at concentrations of 4,000 μM to 0.8 µM were dissolved in 10% DMSO in DPBS. Tested samples were prepared by resuspending an exact mass of freeze-dried powder in DPBS to obtain a range of concentrations from 300 μM to 0.6 µM. DPBS and blank PLGA NPs were used as negative control and WFI as positive control. Samples were distributed at 25 µL per well in a U-bottom 96-well plate. The plate was shaken for 10 min at RT at 750 rpm with a microtiter plate shaker. After incubation, 50 µL of purified sheep red blood cells were added to the samples and incubated for 30 min at RT and 450 rpm agitation. The plate was then centrifuged at 4′000 rpm for 5 min at 20 °C. Some 50 μL of supernatant from the centrifuged plate were transferred into a new flat bottom 96-well plate and shaken 2 min at RT, 450 rpm. Finally, lysis was measured by UV-vis (SYNERGY MIX, BioTek) at a lambda max of 412 nm. The experiment was repeated thrice.

### 2.7 Antigen stability assay

Antigen was stabilized by assessing HA integrity using an HA ELISA assay kit (Sino Biological, China). First, a suspension containing the *in vivo* dose of adjuvants (FA: 33 mg, FB: 19 mg) and WH5N1 1.5 µg in 100 µL of PBS was incubated at 4 °C for 24 h; WH5N1 1.5 µg incubated for 48 h at 60 °C was used as a positive control, and 1.5 µg of WH5N1 alone was used as negative control. ELISA assay was performed following the manufacturer’s protocol. A 96-well half-plate was coated with mouse anti-H5N1-HA-Mono-Ab at a concentration of 0.5 μg/mL in PBS and incubated overnight at 4 °C. WH5N1 1.5 µg incubated for 48 h at 60 °C was used as a positive control. The next day, the plates were washed three times with washing buffer (WB) composed of 0.05% Tween 20 in Tris-Buffer-saline (TBS) buffer; they were then blocked with a 2% BSA in TBS solution for 1 h at RT. After that, the samples were diluted in a 1% BSA in TBS solution and titrated from an estimated concentration of 1.5 μg/mL of WH5N1 to 0.012 μg/mL. Similarly, a calibration curve was prepared with HA concentrations ranging from 5,000 pg/mL to 39 pg/mL. After plating, the samples were incubated under shaking for 2 h at RT. Next, plates were washed thrice with washing buffer and detection antibody, and rabbit anti-H5N1-HA polyclonal was added at a concentration of 1.5 μg/mL and incubated under shaking for 1 h at RT. After three washes with WB, the plates were stained with streptavidin-HRP at 1 mg/mL and incubated for 1 h under shaking at RT in the dark. After three more washes, 25 µL of a TMB A:TMB B (1:1) solution were added to the plate and incubated 10 min exactly before stopping the reaction with 25 µL of 1 M H_2_SO_4_. Eventually, HA degradation was measured by UV-Vis plate-reader (SYNERGY MIX, BioTek) at 450 nm. The experiment was repeated thrice.

### 2.8 *In vitro* cumulative release

Release assays for tomatine and SG101 from PLGA-Chol NP were performed in a PBS-BSA 0.5 mM buffer pH 7 and acetate buffer-BSA 0.5 mM pH 4. For FA, a precise amount of NPs was weighed and suspended in 5 mL of buffer, then incubated at 37 °C under shaking. For FB, a precise amount of NP was weighed and suspended in 27 mL of buffer, then incubated at 37 °C under shaking. At each timepoint (0, 1, 2, 4, 6, 8, 10, 24 h), 500 µL of suspension were recovered and exchanged with fresh buffer. Samples were centrifuged at 22,000 rpm, 4 °C for 10 min, then supernatant was recovered and frozen at −20 °C. Finally, tomatine and SG101 were quantified by LC-MS with an adapted method as described in Section 2.4. In brief, adjuvants were extracted using a methanol–water 9:1 solution, containing solasonine 0.2 μg/mL to precipitate albumin, then centrifuged 10 min at 10′000 x g and filtrated. The column used was a XSelect PREMIER Peptide HSS T3 2.5 µm, 50 × 2.1 mm (Waters) at 40 °C and an injection volume of 2 µL. The mobile phase was composed of 5 mM ammonium carbonate pH 7 (phase A) and isopropanol (phase B). The gradient’s parameters were 10%–80% phase B in 4 min, then 80% phase B for 2.5 min, to then go back to 10% phase B for 2 min with a flow rate of 0.3 mL/min. The temperature of the autosampler was 20 °C. Analyses were performed in positive ESI mode with selected ion monitoring (SIM) with a source temperature of 150 °C. The flow of desolvation gas (N_2_) was 1000 L/h at 450 °C and the cone gas (N_2_) at 10 L/h. The capillary voltage was 3.0 kV. The experiment was repeated thrice.

### 2.9 Mouse experiments

All animal work was completed in accordance with Swiss federal legislation on animal protection (VD3691). Female C57BL/6 mice 6–7 weeks of age were procured from Charles River (US). After 1 week’s acclimatization, the mice were randomly divided into six groups (six mice/group), then immunized intramuscularly (IM) twice at a 3-week interval with a 100 µL suspension (50 µL per leg) of 1.5 µg of WH5N1 plus PLGA-Chol NP FA (100 µg SG101 and 462 µg of tomatine) and FB (100 µg SG101 and 223 µg of tomatine). WH5N1 vaccine alone was used as an unadjuvanted control, and FA and FB NP alone were used as negative controls. Blood samples were collected at days 0, 20, and 42 for antibody titer measurement. At day 43, an antigen boost was performed on mice with 1.5 µg WH5N1. At week 7, mice were euthanized by intraperitoneal injection of pentobarbital, and splenocytes were collected for T cell response analysis.

### 2.10 Immunogenicity measurements: HA inhibition assay

Humoral HA-specific antibodies were quantified using HA inhibition assay following the WHO “Manual for the Laboratory Diagnosis and Virological Surveillance for Influenza” (Organization, 2011). Serum samples were incubated overnight at 37 °C with receptor-destroying enzyme (RDE) at a 1/5 v/v and then plated in a 96-well plate with 1.5% sodium citrate at a 1/10 dilution. Turkey red blood cells (RBC) purchased from Envigo (US) were washed three times with PBS-BSA 0.05% and counted to 5*10^7^ cells/mL. Agglutination in non-treated sera was assessed by two-fold dilution of the serum samples in PBS and incubated 30 min at RT. If RBCs did not settle or were torn in this time, non-specific agglutination was occurring, requiring another RDE treatment. *In vivo* sera samples were then serially diluted 1:1 in V-bottom 96-well plates in PBS, while WH5N1 inactivated vaccine was adjusted to 0.16 HA units/µL and the same amount of standardized antigen was added to each well. Plates were then incubated for 30 min at RT, then 50 µL of RBC were added. After 40–45 min, control RBCs must form a circular shape on the bottom of the wells and tear from the well when the plate is inclined at 90°. HAI titers are the reciprocal of the last serum dilution containing non-agglutinated RBC; a titer of 1:9 was assigned to samples with results below the lowest dilution. HAI assay was repeated twice to assess reproducibility.

### 2.11 Immunogenicity measurements: T-cell response by intracellular cytokine staining (ICS)

C57BL/6 mice spleen was recovered after euthanasia and crushed through a 70 μm cell strainer to isolate splenocytes. These were washed with RPMI supplemented with 1% pen/strep, 20 mM Hepes + 1x MEM non-essential amino acids. Splenocyte isolation was obtained from the interphase of Lympholyte M (Cedarlane, CA) gradient after 20 min of centrifugation at 1,500 rcf without a break. After centrifugation, 10^7^ splenocytes/mL were added to a round-bottom 96-well plate and stimulated with 30 µg HA/mL of WH5N1 inactivated virus and α-CD28 (Pharmingen, NJ) or medium and incubated for 2 h at 37 °C. BD GolgiPlug^®^ (BD Biosciences, CA) at 1/1,000 dilution was added to the wells and incubated overnight at 37 °C, 5% CO_2_ to stop cytokine secretion. Cells were then transferred to a V-bottom 96-well plate and stained with LIVE/DEAD^®^ (Invitrogen, MA) for 15 min at 4 °C. Next, cell surfaces were stained for 20 min at 4 °C with anti-CD3e FITC (Invitrogen), anti-CD4 Pacific Blue, anti-CD8 PerCP/Cy5.5 (Biolegend), and antiCD16/32 (Pharmingen). After washing, cells were fixed and permeabilized with BD Cytofix/Cytoperm (BD Biosciences) for 20 min at 4 °C and then stained with intracellular cytokine antibodies anti-IL2 APC and anti-IFNγ PE (Pharmingen) for 30 min at 4 °C. After that, cells were washed and resuspended in 150 µL PBS for flow cytometry acquisition on a Attune NXT (ThermoScientific, MA). Compensation was performed using compensation beads (Invitrogen). All analyses were performed using FlowJo software 9 (FlowJo LLC, USA). Triplicates were performed for each splenocyte sample.

### 2.12 Statistical analysis

All data are represented as mean ± SD and represent three separate experiments. Hemolysis experiments and *in vivo* results were analyzed by two-way ANOVA with multiple comparisons. Each animal study group is represented by the individual animal titer. All *p* values <0.05 were defined as statistically significant.

## 3 Results

Manufacturing process optimization was performed through testing and selecting various manufacturing methods such as microfluidics, nanoprecipitation, and simple emulsion solvent evaporation. The latter was selected due to the poor selection of solvents compatible with the two adjuvants. Optimization was later effected by testing the best cryoprotectant, tensioactive percentage, and formulation parameters. After the manufacturing method was selected, different concentrations of adjuvants were analyzed, and finally formulations A and B were determined to be the most promising considering the balance between physico-chemical characteristics and toxicity.

Formulations A and B (FA and FB) (composition listed in Table 1) were physico-chemically characterized after freeze drying. Their particle size, distribution, and surface charge were measured after resuspension in WFI by DLS and ELS. Shape and morphology were also determined by SEM imaging. UPLC-MS was used to quantify the immunostimulatory components encapsulated in the formulation, as well as to assess their release profile from PLGA-Chol particles in various media. Given the presence of saponins, known for their hemolytic activities, *in vitro* toxicity was performed on both murine macrophage cell-lines and sheep red blood cells (RBC) to confirm appropriate quenching by the presence of cholesterol in the formulation. WH5N1 antigen stability for 24 h in the presence of adjuvants was assessed to ensure antigen integrity of the vaccine *after formulation with the adjuvants*. An immunogenicity study using mice was performed to evaluate both humoral and cellular responses.

**TABLE 1:** Components’ mass for the formulation of one batch of FA and FB.

Components for 1 batch	FA	FB
PLGA [mg]	19.9	12.9
Cholesterol [mg]	1.13	3.5
Tomatine [mg]	1.13	3.5
SG101 [mg]	0.3	0.5
Sucrose [mg]	40	40

### 3.1 Physico-chemical characterization of PLGA-Chol samples

**FIGURE 2:**
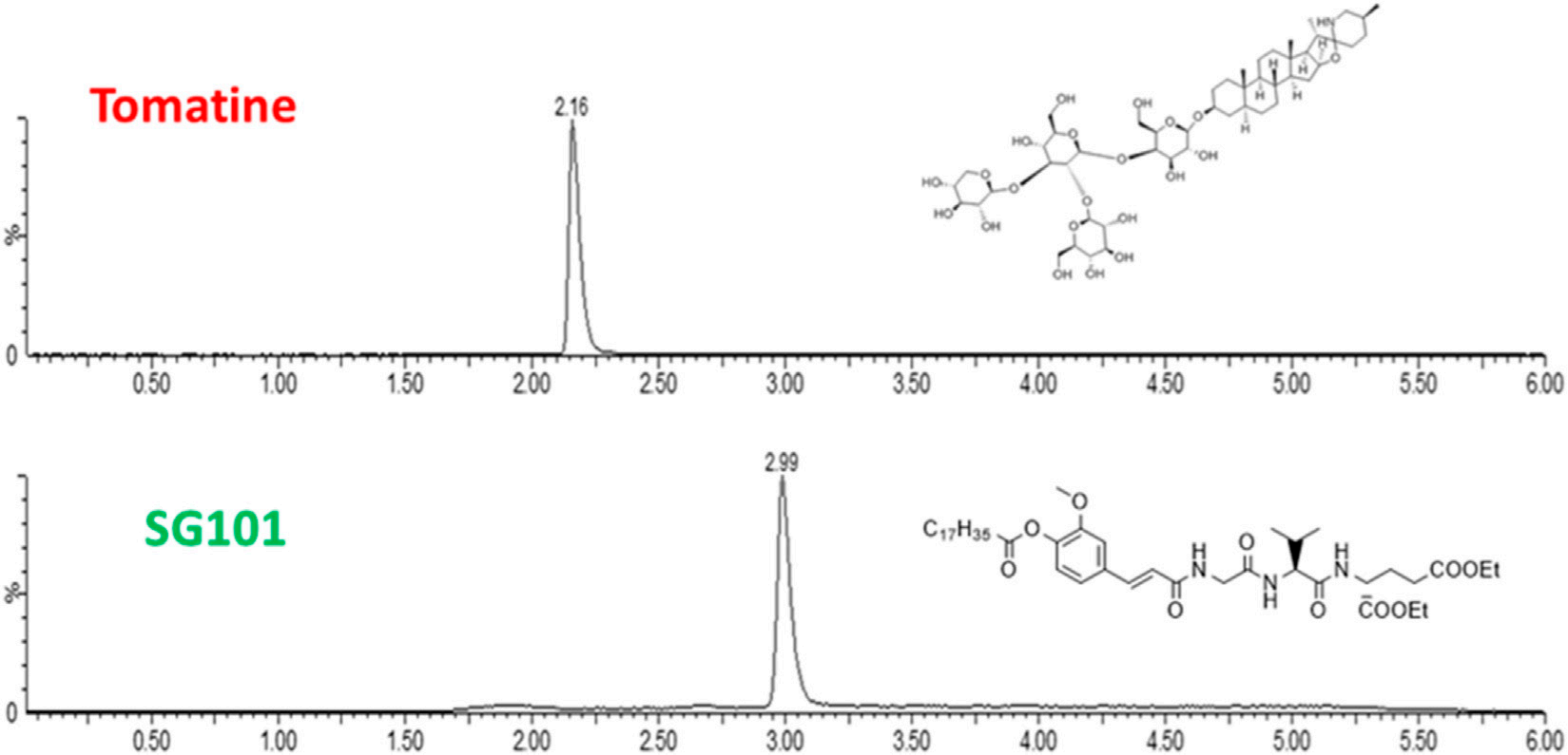
Chromatograms of tomatine and SG101 after LC-MS (ESI+) separation and acquisition. The *X*-axis represents time of retention, and *y*-axis, % of intensity. Quantification performed through peak integration.

While various manufacturing methods were tested, the single emulsion solvent evaporation technique proved to be the most efficient. One of the reasons being the low solubility of both adjuvants. In fact, tomatine is an amphiphilic compound with a cLogP of 0.7, with sufficient solubility in alcohols and tetrahydrofurane (THF), making it ideal for nanoprecipitation manufacture. However, when formulated with cholesterol it forms insoluble complexes that precipitate when using this method. SG101, on the other hand, is a highly hydrophobic compound with a cLogP of 10.26, soluble in DCM. Single emulsion evaporation method allows the solubilization of SG101 in DCM and the encapsulation of tomatine-cholesterol complexes in PLGA through the high shear stress caused by sonication. The first step was to establish a method to quantify adjuvant encapsulation efficacy. In Figure 2, LC-MS (ESI+) chromatograms of tomatine and SG101, together with their chemical structure are presented. Tomatine peak appears at 2.16 min being more hydrophilic than SG101, appearing at 2.99 min. Peak analysis allowed the quantification of the molecules. Limit of detection (LOD) of tomatine was measured at 0.7 ng/mL ang at 1.2 ng/mL for SG101, whereas the respecitive limits of quantification (LOQ) were 2 ng/mL and 4 ng/mL.

**FIGURE 3:**
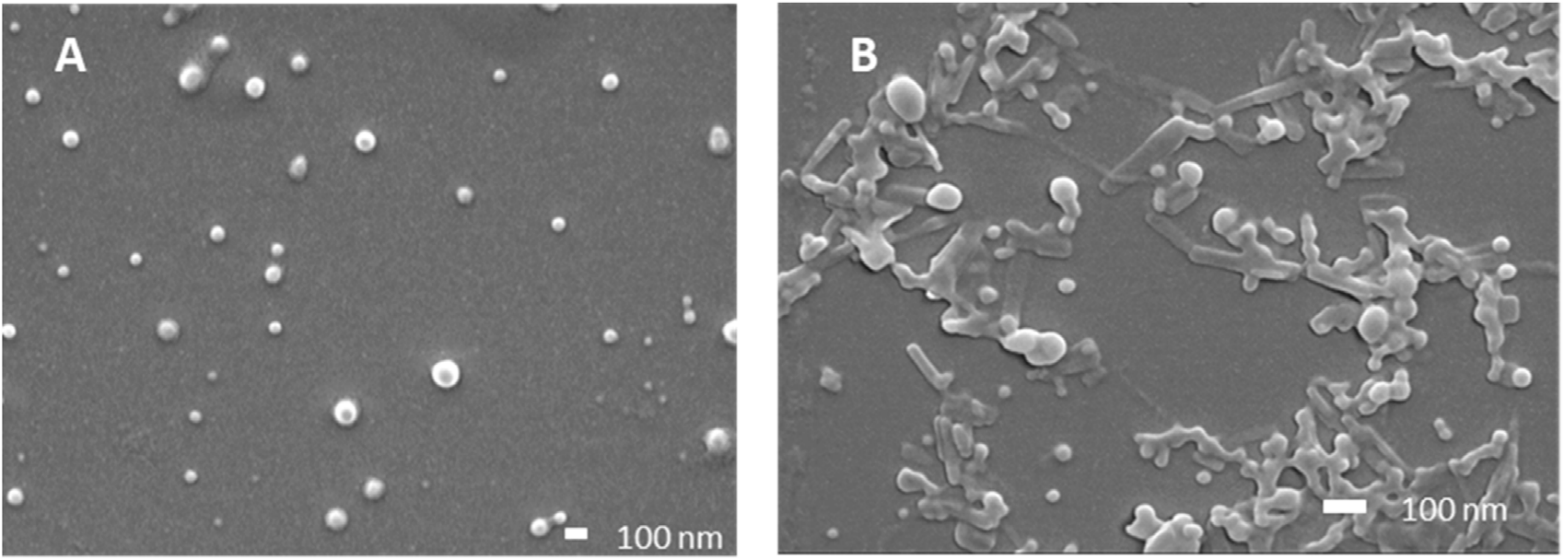
Scanning electron microscopy images of PLGA-Chol samples. **(A)** Formulation **A; (B)** formulation **(B)**. All samples were resuspended in MilliQ water before drying under vacuum. Scale bar is at 100 nm.

Particle size, size distribution, and encapsulation efficacy were then determined (Table 2). FA and FB differed in their drug loading (DL) of SG101, which is higher for FB compared to FA. Tomatine DL after freeze-drying, on the other hand is similar for the two formulations. In fact, FB encapsulation efficacy for tomatine is approximately three times lower compared to FA, even though there is three times more tomatine in FB compared to FA, which makes their final drug loading comparable. Nonetheless, SG101 encapsulation efficacies are similar for the two samples, but FB has almost twice the amount of SG101 in its formulation. FA show smaller particle sizes and a better homogeneity compared to FB. To induce macrophage uptake, nanoparticles need to be below 500 nm (Gustafson et al., 2015), therefore both formulations are in the desired size range. In Figure 3, SEM images of the two formulations are shown. Figure 3A shows a homogeneous sample, with sizes around 100 nm, representing FA. On the other hand, Figure 3B shows spherical particles and elongated crystal-like structures, likely tomatine and cholesterol complexes and PLGA particles. This explains the lower encapsulation efficacy of FB as some of the tomatine-cholesterol complexes were not encapsulated. Major differences in particle size were observed between the DLS and SEM measurements for both images. This was due to the fact that DLS measures the hydrodynamic particle diameter, in terms of intensity, which usually leads to an over-estimation of particle sizes (Bhattacharjee, 2016). Moreover, DLS is meant to measure spherical particles, and is not ideal for crystal-like structures observed in FB images, which also explains the high values for PDI and size.

**TABLE 2:** Physico-chemical characteristics of PLGA-Chol-tomatine-SG101 formulations A (low DL) and B (high DL). Reported are size, PDI, and drug loading of both adjuvants. Data are represented as mean ± SD (n = 3).

Formulation A (low DL)	Average	SD	Formulation B (high DL)	Average	SD
Size [nm]	224.88	32.43	Size [nm]	309.76	45.88
PDI	0.210	0.028	PDI	0.327	0.014
[%]Drug loading tomatine	4.0	0.5	[%]Drug loading tomatine	4.3	0.9
[%]Drug loading SG101	0.9	0.6	[%]Drug loading SG101	1.7	0.3

**FIGURE 4:**
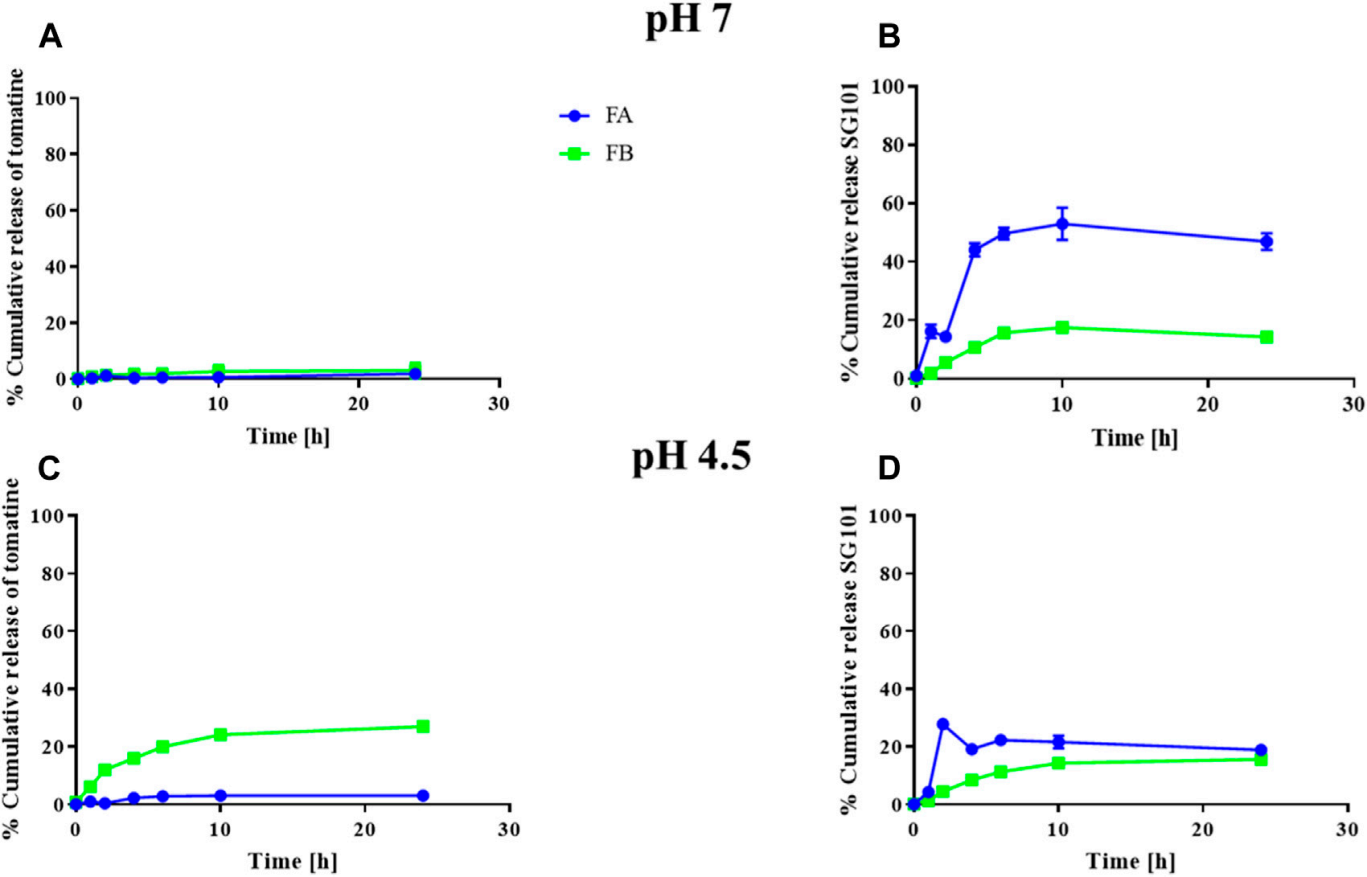
Cumulative release of tomatine and SG101 from PLGA-Chol NP over 24 h. **(A)** Cumulative release of tomatine at pH 7, **(B)** cumulative release of SG101 at pH 7, **(C)** cumulative release of tomatine at pH 4.5, and **(D)** cumulative release of SG101 at pH 4.5. FA is represented in blue, and FB, in green. Results are shown as mean ± SD, n = 3. Results are normalized to 100% corresponding to the total concentration of adjuvant in the batch tested, being: for FA: 0.069 ± 0.006 mg/mL for tomatine and 0.019 ± 0.007 mg/mL for SG101; for FB: 0.005 ± 0.001 mg/mL for tomatine and 0.0008 ± 0.0002 mg/mL SG101.

The cumulative release of tomatine and SG101 from FA and FB were also investigated (Figure 4). NPs were tested at pH 7 and 4.5 in a protein-rich medium to compare the *in vitro* release of the molecules in a physiological buffer and in a lysosomal-like medium, respectively (Li et al., 2019). In Figure 3A, we observed a release of tomatine close to 0% for both FA and FB formulations at pH 7. A decrease in pH to 4.5 increased the release of tomatine from FB but not from FA. It is likely that the cholesterol–tomatine complexes observed in Figure 3.B were degraded faster by the lower pH. To achieve complete release of tomatine, the full degradation of the PLGA-Chol would need to be evaluated, which would be expected after more than 96 h (Supplementary Figure S1). The release of SG101, on the other hand, is faster in FA than in FB and at pH 7 rather than pH 4.5. The pH of the release medium has a more pronounced influence on low loading formulation (FA), while for high loading formulation (FB) the pH appears to have a lesser influence on the release profile. After 24 h at pH 7, 47.0% ± 2.8% and 14.3% ± 1.7% of SG101 are released in the medium for FA and FB, respectively. At pH 4.5, only 18.9% ± 0.9% and 15.7% ± 1.1% were released.

### 3.2 *In vitro* toxicity assessment of PLGA-Chol formulations

**FIGURE 5:**
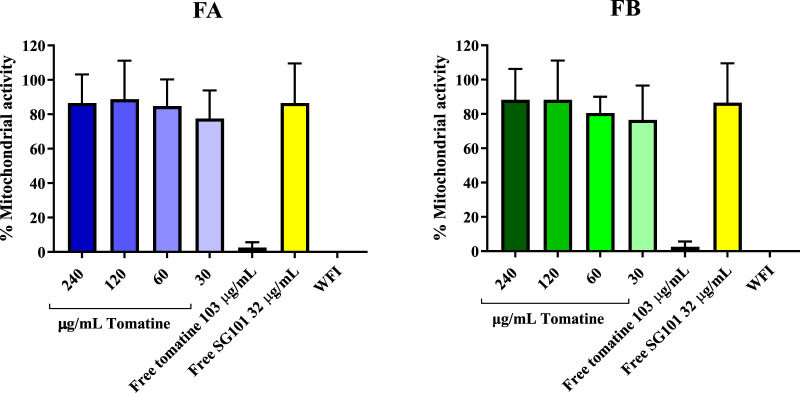
Mitochondrial activity of RAW 264.7 cells after incubation with formulation A (blue) and formulation B (green) at various concentrations of tomatine for 4 h at 37°C, 5% CO_2_. Un-encapsulated tomatine at a concentration of 103 μg/mL and WFI were used as positive controls. SG101 at concentration of 32 μg/mL was used as the negative control. Tomatine 103.4 μg/mL corresponds to the minimum dose at which the free drug is 100% hemolytic (Supplementary Figure S2). SG101 32 μg/mL corresponds to the average concentration of SG101 found in the particles for the same amount of tomatine tested as control. Data are normalized to untreated cell results, having an OD value of 1,353 ± 0.558. Data are represented as mean ± SD (n = 4).

**FIGURE 6:**
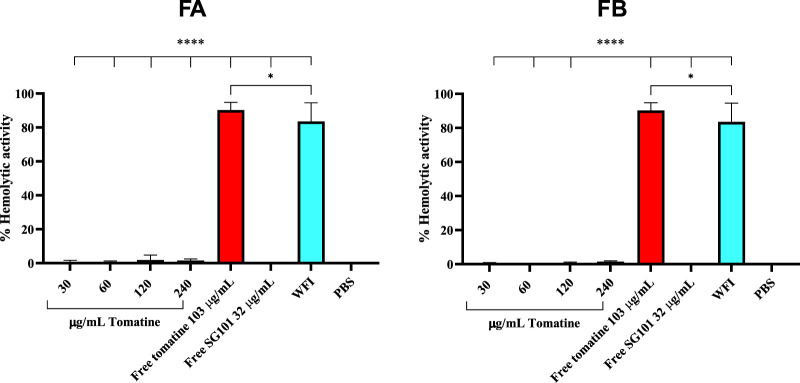
Hemolytic activity of defibrinated sheep red blood cells after incubation with formulation A (blue) and formulation B (green) at various concentrations of tomatine. Un-encapsulated tomatine at a concentration of 103 μg/mL and WFI were used as positive controls. SG101 at a concentration of 32 μg/mL and cells treated with PBS were used as negative controls. Tomatine 103.4 μg/mL corresponds to the minimum dose at which the free drug is 100% hemolytic (Supplementary Figure S2). SG101 32 μg/mL corresponds to the average concentration found of SG101 in the particles for the same amount of tomatine tested as control. Data are represented as mean ± SD (n = 3). **** indicates *p* < 0.0001, and * indicates *p* < 0.05 when comparing the WFI group with other groups. Tomatine has higher hemolytic activity than WFI. Here, 100% of hemolytic activity is considered (OD (sample)-OD (negative control)/OD (positive control)), considering that OD average values of negative control (untreated cells) are 0.173 ± 0.009 and those of positive control (WFI) are 1,568 ± 0.102.

Saponins are known to complex with cholesterol present in the cell membrane, thereby causing cell disruption (Wang et al., 2020). To neutralize this phenomenon, it is necessary to incorporate cholesterol in the formulations. As a means of confirming the quenching of saponins, hemolysis assays or *in vitro* toxicity assay using RAW 264.7 cells can be used. As shown in Figure 5, the mitochondrial activity of murine macrophages incubated for 4 h with FA and FB was measured at various concentrations of tomatine. The graph shows mitochondrial activity above the 80% threshold of viability for all formulations tested, whereas tomatine alone shows mitochondrial activity values close to WFI, suggesting cell toxicity. SG101 alone, as expected, does not seem to influence cell viability. These results indicate that the addition of cholesterol to the PLGA formulation neutralized the lytic properties of tomatine, rendering the adjuvant suitable for delivery by intramuscular injection. These results were confirmed by a hemolysis assay on RBCs (Figure 6), where a similar trend is observed. In fact, FA and FB show hemolytic activity close to 0%, whereas tomatine alone is significantly more hemolytic than the positive control WFI.

**FIGURE 7:**
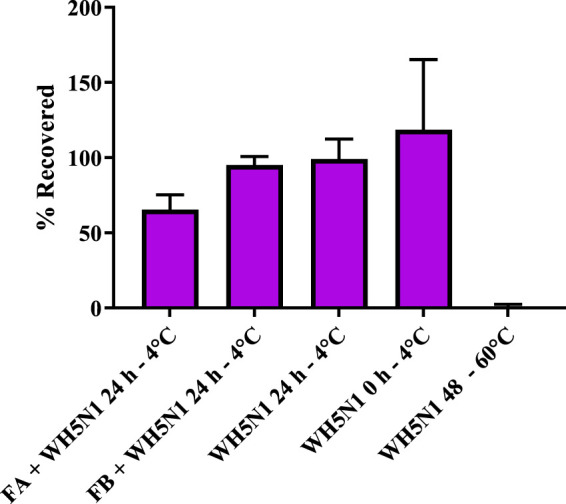
Antigen stability (WH5N1- 1.5 µg) after incubation with formulations A (33 mg) and B (19 mg) at the *in vivo* dose in 100 µL of PBS for 24 h. WH5N1 antigen, 1.5 µg, incubated for 48 h at 60 °C was used as the positive control. An amount of 1.5 µg WH5N1 incubated in PBS for 24 h and fresh antigen were used as negative control. Data are represented as mean ± SD (n = 3). Here, 100% is determined as 1.5 µg WH5N1 recovered after assay.

To ensure that the formulation is compatible with the antigen, it is important to evaluate antigen integrity at 2 °C–8 °C. This was assessed by resuspending the dry nanoparticles in PBS together with WH5N1 antigen at the intended *in vivo* dose and by incubating the suspension for 24 h at 4 °C before estimating HA concentration using an ELISA HA quantification assay. The percentage of HA recovered after incubation is shown in Figure 7, where results are normalized to incubation of antigen alone in PBS for 24 h at 4 °C. FB shows 95% ± 5.8% of HA recovered, suggesting no antigen degradation. Some HA instability was observed for the formulation containing FA, with a recovery of only 65.5% ± 9.7%. This confirmed short-term stability, giving us confidence to prepare the formulations a maximum of a few hours before administration in the *in vivo* studies.

### 3.3 *In vivo* immunogenicity of PLGA-Chol formulations

**FIGURE 8:**
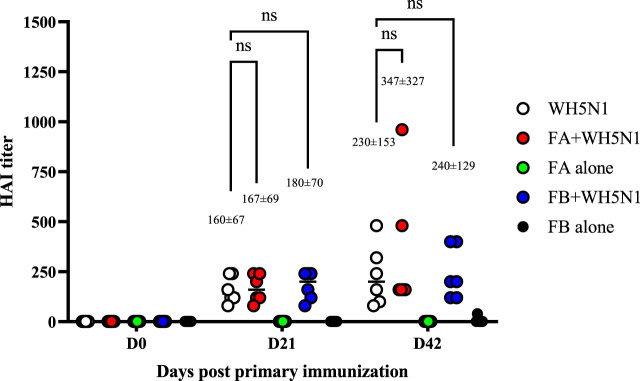
Antigen-specific antibodies measured by HA inhibition assay in C57BL/6 mice after a prime and boost i.m. injection of adjuvanted vaccine at days 0 and 21. WH5N1 was injected at a dose of 1.5 µg per 50 μL in combination with PLGA-Chol FA formulation or with FB formulation (both containing tomatine and SG101). Both FA and FB were also injected i.m. with PBS as adjuvant-only controls. Mouse sera were recovered at days 0, 21, and 42. Results are presented as individual mouse titer (circle) and as mean (bar) (n = 6). HA inhibition assay was repeated twice. Two-way ANOVA with multiple comparison was used for statistical analysis. ‘Ns’ indicates no statistical difference.

Adjuvant doses for *in vivo* studies were based on Guzelj et al. (2021) on the adjuvanticity of various NOD2 agonists, among which SG101 was the most prominent. The dose of PLGA-Chol NP was measured to carry a dose of 100 µg of SG101 for each mouse, being 33 mg for FA and 19 mg for FB. C57BL/6 mice were immunized with 1.5 µg of WH5N1 antigen at days 0 and 21. To check their antibody titers, sera were collected at days 0, 21 and 42 and analyzed by HA inhibition assay. Control groups were injected with NP alone and PBS, without antigen. Figure 8 shows the humoral response of mice at days 0, 21, and 42. First and foremost, no significant difference in antibody titer was demonstrated between mice immunized with inactivated vaccine alone or with adjuvanted vaccine. However, when taking a closer look at the mean values, slightly enhanced activity is observed in adjuvanted vaccines. At day 21, the mean titer of WH5N1 alone is 160 ± 67, whereas it is respectively 167 ± 69 and 180 ± 70 for FA and FB. Indeed, the difference in this case is almost imperceptible. At day 42, on the other hand, FA adjuvanted vaccine showed a mean titer of 347 ± 327 compared to 230 ± 153 of antigen alone. The HAI titer measured for the group receiving FB was 240 ± 129, comparable to antigen alone. FA and FB formulations alone, without antigen, did not induce antibody responses, as expected.

**FIGURE 9:**
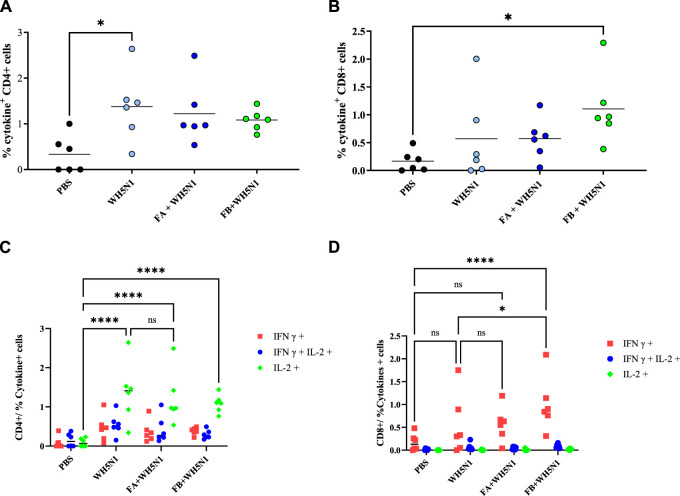
Antigen-specific T-cell responses induced by PBS, WH5N1, adjuvanted WH5N1 with FA, and WH5N1 plus FB, after antigen boost 1 week before sacrifice. Splenocytes were collected and stained to quantify by FACS CD4, CD8, and intracellular cytokines IL-2 and IFNγ secreted within the cells. **(A)** Total CD4 + T cells positive to cytokine secretion; **(B)** total CD8 + T cells positive to cytokine secretion; **(C)** CD4 + T cells positive to IFNγ, IL-2, or both cytokines after signal subtraction with unstimulated cells; **(D)** CD8 + T cells positive to cytokines, after signal subtraction with un-stimulated cells. Each dot corresponds to an individual mouse (N = 6); mean is presented for every group. Two-way ANOVA with multiple comparison was used for statistical analysis. Ns show no presence of statistical difference. **** indicates *p* < 0.0001; * indicates *p* < 0.05; ns indicates no presence of statistical significance.

T cell response was also investigated in mice after spleen collection. Figure 9 shows the results obtained after cell sorting of CD4^+^ and CD8^+^ T cells as well as those showing positive for cytokine secretion. Figure 9A and B show total positive CD4 and CD8 cells. CD4 showed no major differences among the four groups tested, except for antigen alone *versus* PBS, where a significant (*p* > 0.05) increase in CD4 production was observed. However, we can see a trend of increased cell production for all treated samples. The trend observed for CD8 is somewhat similar, although it is the adjuvanted FB + WH5N1 group that is significantly increased compared to the control. It also shows an increased response compared to WH5N1 alone, although the difference is not significant. Nonetheless, it is interesting to observe intracellular cytokine expression (Figure 9C, D) after subtraction of the signal for unstimulated cells. Expression of IL-2 in the three treated groups (WH5N1, FA + WH5N1, and FB + WH5N1) shows a significant increase compared to mice treated with PBS alone, suggesting an enhancement of intracellular cytokine production under antigen exposure. Adjuvanted vaccines, however, failed to increase expression in CD4 T cells but did not prevent WH5N1 acting on T cells. Despite this, IFNγ CD8 expression is significantly higher in mice treated with FB adjuvanted vaccine (FB + WH5N1) and the control group (WH5N1).

## 4 Discussion

The role of tomatine as a vaccine adjuvant was mainly investigated by Morrow and colleagues in the early 2000s, but information about the mechanism underlying the effect and dosage necessary for adjuvanticity is still lacking (Morrow et al., 2004; Yang et al., 2004; Heal and Taylor-Robinson, 2010; Raj et al., 2014). Moreover, little focus has been dedicated to the development of a proper delivery system for such adjuvants. Exceptions are the use of cholesterol, required for saponin administration, and gold nanoparticles for cholesterolemia quantification (Raj et al., 2014; de Groot and Müller-Goymann, 2016). NOD2 agonist SG101, on the other hand, is a compound that was synthetized for the first time a few years ago in Prof. Jakopin’s laboratory; although it proved to enhance immunogenicity against OVA by liposomal delivery, information on its effects is yet to be discovered as studies on model antigens fail to show antigen-dependent challenges (Guzelj et al., 2021). Overall, the co-delivery of such molecules in a hybrid nanoparticle presented important challenges from the start of this project. First, we encountered the issue of poor adjuvant solubility. Tomatine, like all saponins, is an amphiphilic molecule, generally soluble in alcohols and tetrahydrofuran but insoluble in water. When paired with cholesterol, tomatine creates an insoluble complex that can create aggregates visible to the naked eye if not controlled during manufacture (Bailly, 2021; Zhao et al., 2021). On the other hand, SG101 is a low molecular weight, highly lipophilic drug with a cLogP of 10.26; it has a very low solubility range and is insoluble in alcohols. A delivery system suitable for lipophilic drug delivery was needed, and hybrid PLGA cholesterol particles were especially chosen given their ease of lyophilization. However, methods such as microfluidics or nanoprecipitation were not suitable for their manufacture, given the low solubility of SG101 in water-miscible solvents and the formation of tomatine–cholesterol complexes. Single emulsion was chosen because of the solubility of SG101 in dichloromethane, and the use of sonication helped break and incorporate saponin–cholesterol aggregates, achieving a homogenous nano-scale size (Van de Ven et al., 2011). This study determined the appropriate conditions for freeze-drying by sublimating the liquid of the nanoparticle formulation. This was advantageous for formulation (resuspension) at the desired dose but also to ensure the storage stability of the formulation. Longer shelf stability for dried nanoparticles may also improve their resistance to temperature changes (Preston and Randolph, 2021; Muramatsu et al., 2022). This type of thermostability has, however, not yet investigated. The advantage of an adjuvant with thermostable characteristics is that storage is suitable in infrastructure conditions that are not suited for a sub-zero cold chain. The delivery material PLGA was selected with this objective in mind. Other than being a highly versatile polymer which is safe and suitable for the encapsulation of lipophilic molecules, freeze-drying PLGA polymer particles is well-documented and generally successful using the right cryoprotectants (Fonte et al., 2016a; Fonte et al., 2016b). Nanoparticle sizes ranged from 200–300 nm—quite large for drug delivery. PLGA nanoparticles are not expected to migrate from the site of injection after delivery and will be likely be taken up by local APCs. It was in fact shown how the size of particulate adjuvants is crucial in antigen interaction and APC uptake. Particles larger than 250 nm were shown to enter dendritic cells by micropinocytosis, thus ensuring adjuvant interacts with intracellular receptor after internalization (Autenrieth and Autenrieth, 2009).

The formulations were assessed for cytotoxicity, particularly hemolytic activity. The latter is a relevant characterization required due to the inherent lytic properties of saponins like tomatine (Sudji et al., 2015). As part of its lytic behavior, tomatine has a high affinity with cholesterol. This feature can be used in formulating tomatine in combination with excess cholesterol, which can reduce the cytotoxicity of the saponin without impairing its adjuvancy (Keukens et al., 1995; Stine et al., 2006). Supplementary Figure S2 shows the necessity of adding cholesterol to the formulation to hinder hemolytic activity, which was confirmed in Figures 5 and 6, demonstrating no cytotoxicity of the formulations manufactured. The high affinity of tomatine with cholesterol is also found in the cumulative release results (Figure 6). In fact, saponins’ hemolytic activity is caused by their affinity for cholesterol, and thus they have a tendency to bind to cells’ membrane cholesterol and disrupt cells. This is why cholesterol is required in a saponin formulation and why toxicity in hindered (the ratio necessary for quenching has been evaluated as 1:1) (Caillet et al., 2010; Harrington et al., 2021). The cumulative saponin release over 24 h was, in fact, lower than 5% for FA both at pH 7 and 4.5. It is increased in FB formulation to around 20%, probably due to the lower quantity of PLGA present in the formulation than in FA, with tomatine–cholesterol complexes more exposed to the acidic medium. FA shows higher release of SG101 at pH 7 before NP uptake, and the NOD2 receptor is found intracellularly, besides very low tomatine at any pH. FB has a comparatively better overall release of the two drugs at pH 4.5, suggesting better intracellular receptor targeting. The density of the hybrid nanoparticle will likely also hinder SG101 release, as its release is also incomplete after 24 h and is lower at pH 4.5 than 7; the release profile obtained is a typical type II kinetic release profile. The size of drug particles is a critical element in determining the release kinetics of a drug from PLGA. Our SEM results indicate that tomatine–cholesterol forms large complexes, especially for the FB formulation. It appears likely that SG101, given its hydrophobic nature, is also partly included in the complex through hydrophobic bonds (Xu et al., 2017).

In Supplementary Figure S1, the degradation of FA at pH 4.5 is observed to be over 96 h, while the PLGA nanoparticles lose their characteristic shape between 24 h and 48 h. Rather than degrading and developing the typical porous morphology of PLGA, the particles tend to aggregate and form a bulk structure, which might hinder the release of adjuvants in the medium (Siegel et al., 2006; Lanao et al., 2011). Moreover, *in vitro* cumulative release was performed in BSA-rich media to increase adjuvant solubility and mimic the biological medium, whereas SEM images (Supplementary Figure S2) have been acquired in water at pH 4.5; thus, nanoparticle degradation in a physiological medium can greatly differ.

One of the aims of this project was to investigate and formulate novel adjuvant combinations capable of increasing cytotoxic T cell response when combined with inactivated influenza vaccine. Research has shown that strong antigen-specific CD8 T cell responses enhance the possibility of developing cross-reactive protection in influenza (Weinfurter et al., 2011; Sridhar et al., 2013; Soema et al., 2015).

Saponins and QS-21 have been widely used in combination with other immunostimulants such as TLR4 agonist MPL^®^ in the AS01 adjuvant. In particular, synergy between a saponin and TLR4 agonist has been observed to increase the adaptive immune response (Coccia et al., 2017).

Here we propose the combination of tomatine and SG101, a NOD2 agonist. Not only is this a more sustainable source of saponin than QS21 and a safer analog of MDP but there is also a potential for immunomodulatory synergy when formulated in a suitable carrier. As a derivative of MDP—the smallest naturally occurring immune stimulatory component of cell walls from *Mycobacterium*—SG101 has similar immunostimulatory effects as natural MDP, with the additional advantage of being more hydrophobic to facilitate its passage through the cell wall and not causing excessive reactogenicity. Potential synergy can be achieved where SG101 acts as a nonspecific immunomodulator by activating macrophages and monocytes with secretion of pro-inflammatory cytokines (TNF-a, IL-1, IL-6), enhancing antigen presentation, cytokine production, and the recruitment of immune cells. Saponins, on the other hand, are known for their ability to induce the release of pro-inflammatory cytokines and promote the activation of antigen-presenting cells (Wang et al., 2020).

Such synergy was not directly observed with the hybrid nanoparticle formulations investigated in this project. The *in vivo* study conducted in C57BL/6 mice did not produce the desired immunogenicity, as the titers observed for both antibodies and T cells in groups immunized with FA and FB plus the antigen were generally similar to those obtained by vaccinating mice with the antigen alone. FA and WH5N1 alone did not provide any improvement in expression compared to control. However, a positive trend in antibody response was observed as well as, more importantly, a significant enhancement of IFNγ expression in CD8 for the FB-WH5N1 group. The adjuvant value of the novel combination might be better highlighted by using split influenza antigens rather than WIV antigen, which contains intrinsic immunostimulants such as TLR9 ligands (Kostinov et al., 2018).

Further formulation optimization and, in particular, dosing studies for each component would be required to better confirm and characterize the potential synergy. In particular, we expect that the dose of tomatine may still be suboptimal in this formulation. Tomatine was, in fact, only scarcely used as an adjuvant, and generally very high doses of saponins were used in animal studies—not ideal when dealing with the development of a pandemic vaccine (Heal and Taylor-Robinson, 2010). Moreover, the dose was not specified when formulated with OVA and the bacterium *Francisella tlanensis* (Sun et al., 2009). The dose of SG101 component was selected based on the more recent studies conducted with SG101 in combination with ovalbumin (OVA) antigen immunization studies (Guzelj et al., 2021). Although other studies may provide an indication of the dose to use, there is always a strong influence of the antigen; in these hybrid NP formulations, the release kinetics may also greatly influence immunogenicity. This study aimed to improve release kinetics by using a PLGA co-polymer. A co-polymer-based carrier would result in more porous particles, thereby supporting the release of immunomodulators and, therefore, immunogenicity. Particle degradation and slow release could prevent immunomodulators from activating downstream pathways and inducing the desired immune responses.

Tomatine has a high affinity with cholesterol, which can also mean that, due to the presence of cholesterol in the hybrid NPs, the tomatine is not efficiently released. A saponin other than QS21 with a lower affinity to cholesterol would be an interesting alternative. The saponin solanine, if incorporated into the hybrid NP—a saponin-based immunomodulator—could have better release kinetics than tomatine and more effectively induce the desired adjuvant effects (Keukens et al., 1996).

## 5 Conclusion

Among the main advantages of combination adjuvants is the possibility of using the synergy of two different compounds to modulate immune response. In this study, we formulated novel hybrid PLGA nanoparticles containing the immunomodulators tomatine and SG101, a saponin and NOD-2 ligand, respectively. These studies highlight several challenges and considerations for a formulation strategy for combination adjuvants, particularly with regard to solubility issues and having the appropriate manufacturing and characterization method to evaluate the high compound loading required.

The preliminary immunogenicity results in mice reported here with a relevant pandemic influenza strain (whole H5N1) showed a significant enhancement of IFNγ expression in CD8^+^ T cells for formulation with the highest loading of tomatine and SG101. These results are promising and should be followed up with further preclinical studies to assess the contribution of tomatine alone, SG101 alone, and the best ratio between the two immunomodulators. Moreover, enhanced physico-chemical studies on formulation stability need to be assessed to determine the exact shelf-life of the particles at different temperatures.

## Data Availability

The original contributions presented in the study are included in the article/Supplementary Material; further inquiries can be directed to the corresponding author.
