# Corrigendum: Preliminary results on novel adjuvant combinations suggest enhancement immunogenicity of whole inactivated pandemic influenza vaccines

**DOI:** 10.3389/fddev.2024.1519969

**Published:** 2024-12-16

**Authors:** Allegra Peletta, Aurélie Marmy, Samo Guzelj, Alcidia Ramos Barros, Žiga Jakopin, Gerrit Borchard

**Affiliations:** ^1^ Section of Pharmaceutical Sciences, Institute of Pharmaceutical Sciences of Western Switzerland (ISPSO), University of Geneva, Geneva, Switzerland; ^2^ Faculty of Pharmacy, University of Ljubljana, Ljubljana, Slovenia

**Keywords:** pandemic influenza vaccine, adjuvant, hybrid PLGA nanoparticle, tomatine saponin, NOD2 agonists, formulation, immunogenicity

In the published article, there was an error in the **Funding** statement. “Swiss National founding, grant no: 20 IZCOZ0_182947” was included incorrectly. Instead, it should appear as “Swiss National Science Foundation (SNF), grant no: IZCOZ0_182947.” The correct **Funding** statement appears below.

