# Supplementary material for: Preliminary results on novel adjuvant combinations suggest enhanced immunogenicity of whole inactivated pandemic influenza vaccines
Source: Front Drug Deliv. 2024 Jul 16;4:1382266. doi: 10.3389/fddev.2024.1382266 (PMC12363304; doi:10.3389/fddev.2024.1382266)
Supplement: Supplementary file 1 [file DataSheet1.docx]

**Supplementary Fig. 1: Degradation of FA at pH 4.5**

**
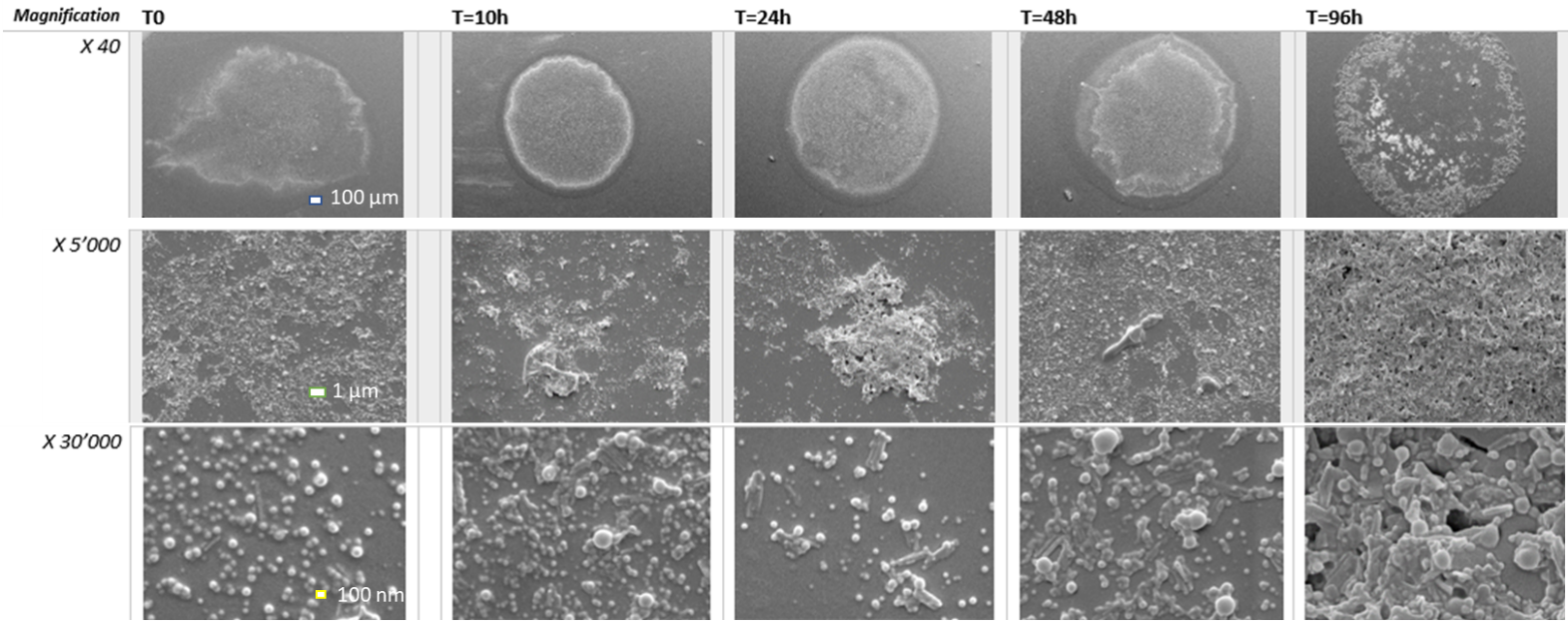
**

**Figure S1**: SEM images of FA degradation at pH 4.5 over 96 h at different magnifications. 96 h was considered as degradation endpoint as NPs are highly disrupted and the polymer aggregated. Scale bars are at 100 µm at X 40 magnification, 1 µm at X 5000 and 100 nm at X 30000.

**Supplementary Fig 2: Influence of PLGA and Cholesterol on Tomatine’s hemolytic activity**

**Figure S2**: Titration of tomatine’s hemolytic activity on sheep RBC after incubation with tomatine alone, tomatine formulated with PLGA and tomatine formulated with PLGA-Chol (1:1 tomatine-Chol ratio). Tomatine alone was dissolved in a 10% DMSO solution and added to the cells. PBS was used as a negative control and WFI as positive control.
